# Balancing promise and concern in AI therapy: a critical perspective on early evidence from the MIT–OpenAI RCT

**DOI:** 10.3389/fmed.2025.1612838

**Published:** 2025-05-22

**Authors:** Yaakov Ophir, Refael Tikochinski, Zohar Elyoseph, Yaniv Efrati, Hananel Rosenberg

**Affiliations:** ^1^Department of Education, Ariel University, Ariel, Israel; ^2^Centre for Human-Inspired Artificial Intelligence (CHIA), University of Cambridge, Cambridge, United Kingdom; ^3^Experimental Psychology Department, University College London, London, United Kingdom; ^4^Faculty of Education, University of Haifa, Haifa, Israel; ^5^Faculty of Education, Bar-Ilan University, Ramat Gan, Israel; ^6^The Moscowitz School of Communication, Ariel University, Ariel, Israel

**Keywords:** AI therapy, digital mental health, technological panic, mental health innovation, AI in mental health

## Abstract

The emergence of AI therapy chatbots has the potential to reduce the widening gap between the huge demand for psychological support today and the limited availability of professional care. However, some scholars and clinicians are warning that the integration of these chatbots could paradoxically lead to negative outcomes, such as dependence, loneliness, and depression. Recently, a joint research team from MIT and OpenAI conducted a four-week Randomized Controlled Trial (RCT), reporting that “*while participants on average were less lonely after the study… extended daily interactions with AI chatbots can reinforce negative psychosocial outcomes*”. Considering the substantial public and academic attention that followed the preprint publication of this RCT, it is crucial to examine the strength of the evidence and the validity of its interpretation before drawing firm conclusions. In this commentary, we offer a careful and appreciative review of this well-designed and timely study. Nonetheless, we argue that due to key analytical limitations, the findings do not substantiate claims of harmful effects. Given the transformative potential of AI-based interventions, we urge caution in interpreting early findings and offer practical guidance for future research.

## Introduction

The global burden of mental health disorders remains a pressing concern, particularly in the wake of the COVID-19 crisis (1, 2). Despite decades of research and clinical advancement, rates of mental health diagnoses and psychopharmacological prescriptions continue to rise (3–5). Against this backdrop, many have placed hope in Artificial Intelligence (AI) as a potential game-changer in mental health care.

Psychologically oriented AI chatbots, designed to function as sensitive personal companions, offer a promising avenue to expand access to psychological support—particularly for individuals experiencing mild symptoms or everyday emotional struggles. Such tools might help alleviate some of the pressure on overburdened mental health systems (6).

However, the prospect of “machine-based” psychological assistance also elicits understandable skepticism. Critics argue that AI systems cannot replicate the authentic empathy and human connection that are widely regarded as central to the effectiveness of traditional psychotherapy (7, 8). Others raise concerns about the potential harms of these technologies, including the risk of emotional overdependence or even addictive use, which may reduce real-world social interaction and exacerbate feelings of loneliness (9, 10).

To explore these concerns empirically, a research team from MIT and OpenAI conducted a longitudinal randomized controlled trial (RCT), investigating the psychosocial effects of interacting with AI chatbots over the course of 4 weeks (11). Participants were randomly assigned to engage with chatbots featuring different modalities (text or voice) and conversational styles (open-ended, non-personal, or personal). Psychosocial outcomes—including loneliness, emotional dependence, socialization with real people, and problematic use—were assessed both before and after the study.

The authors present a rich and detailed set of analyses, incorporating multiple interaction effects and subgroup comparisons. As might be expected from such a multifaceted design, the resulting findings are complex and at times challenging to interpret. The discussion section reflects a thoughtful effort to engage with this complexity. Nevertheless, the authors open their discussion with a concise summary statement that appears to crystallize the study’s central takeaway—a framing that, in our view, merits closer scrutiny:

“Our study results show that while participants on average were less lonely after the study—especially after interacting with an engaging voice-based chatbot—extended daily interactions with AI chatbots can reinforce negative psychosocial outcomes such as decreased socialization.”

Naturally, these findings drew considerable public and academic interest. Although the study has been shared publicly as a preprint, prior to peer review, its conclusions—particularly regarding potential harms—have already been widely circulated in prominent media outlets such as *Bloomberg* and *Forbes* (12–15). Given the influence such early reports can have on public perception and policy discussions, and considering the broader implications for the development and adoption of AI-based psychological tools, we believe that a careful and balanced examination of the study’s methods and interpretations is both timely and warranted.

## A close inspection of the MIT–OpenAI RCT

Responding to this need, we now take a closer look at Fang et al.’s RCT. While the study offers a timely and ambitious contribution to the emerging field of AI-based mental health interventions, several aspects of its design and interpretation merit further scrutiny. In the following section, we examine key methodological features of the trial—including the definition of control conditions, the modeling of usage effects, and the interpretation of effect sizes—that complicate strong conclusions about psychosocial harms. Our aim is to clarify what the data do and do not support at this early stage of research.

### What counts as a proper placebo?

One of the most fundamental questions in this type of research is: what counts as an appropriate placebo? A traditional waiting-list control group would not suffice here, as it would not allow us to distinguish between the effect of simply interacting with an AI chatbot and the effect of engaging with a chatbot in a personal, emotionally meaningful way. From the structure of the study, it seems the authors recognize this point. However, the chosen reference group in their regressions—the *open-ended* condition—may not serve as an ideal placebo. Because participants in this condition could discuss both personal and non-personal topics, it blurs the line between intervention and control. A stronger internal placebo comparison would be the *non-personal* condition, where conversations were explicitly kept impersonal.

### Mixed signals in the reported outcomes

This makes it difficult to interpret the findings with precision. Nonetheless, when we examine Figure 6 and its underlying regression results in Table 6 (Appendix N), a somewhat surprising pattern emerges: there appears to be a slight advantage for the *personal* (i.e., intervention) condition. While the authors report a significantly more moderate decline in socialization in the *non-personal* (placebo-like) group, this advantage is not reflected across the other outcomes. In fact, for loneliness, emotional dependence, and problematic use, the visual trends point to *steeper deterioration* in the *non-personal* condition—even if these effects are not statistically significant in two of the three outcomes. Taken together, Figure 6 does not offer strong support for the idea that the placebo condition is more benign than the intervention; if anything, the opposite appears to be true.

### The problem of usage duration and reverse causality

More importantly, the study’s attempt to explore the effects of daily usage duration is commendable but methodologically limited. Because *duration was not manipulated* as part of the experimental design, it remains a naturally varying covariate. It is highly plausible—if not likely—that individuals who were more lonely or emotionally vulnerable to begin with were those who used the chatbot more frequently, particularly in the personal condition.

### Modeling change: time 2 is not enough

This key methodological issue could have been partially addressed by modeling change scores (i.e., Time 2 minus Time 1) as the dependent variables, rather than modeling “*the final values of loneliness, socialization, emotional dependence, and problematic use measured at week 4…, controlling for their respective initial values measured at the start of the study*” (p. 4). Alternatively, the authors could have retained the longitudinal time variable (“Week”)—which appeared in their mixed-effects models in Table 3—in the subsequent regression analyses presented in Tables 4 through 6. These approaches would have enabled a more direct and transparent examination of within-person change over time.

In the absence of such adjustments, a competing interpretation remains: that participants with greater initial distress were simply more inclined to engage with the chatbot more frequently. For example, consider a participant named Jonny, who began the study with the highest possible score on the UCLA Loneliness Scale (which includes eight items scored from 1 to 4). Although Jonny may have experienced a meaningful improvement over the course of the study—say, a reduction from 32 to 22—he would likely still report higher loneliness at Time 2 than other participants. In the current modeling approach, Jonny’s high final score would be explained mostly by his baseline loneliness (which had a strong predictive value, *β* = 0.877***), yet his daily usage of the chatbot would still be statistically associated with that same Time 2 score, not with his improvement.

In other words, by modeling Time 2 rather than the change from Time 1 to Time 2, the regression cannot distinguish between outcomes caused by chatbot use and pre-existing levels of distress that led to greater use in the first place. This opens the door to reverse causality and renders any interpretation of “dose–response” effects speculative at best.

For this reason, the main takeaway from this study should center on the primary effects reported by the authors—rather than on exploratory interaction terms—namely that “*participants on average were less lonely after the study, especially after interacting with an engaging voice-based chatbot*.” This finding, modest as it may be, aligns with long-standing clinical thinking, which views emotional expression, personal disclosure, and the experience of being heard—even in brief or technologically mediated interactions—as potentially beneficial (16, 17). These assumptions have guided psychological theory and practice for decades, and it would be surprising if they were suddenly invalidated simply because the source of empathy was algorithmic.

Moreover, the usage levels in this study were quite low: participants spent on average only 5.32 min per day interacting with the chatbot, and even the most engaged participant averaged less than 28 min daily. Against this backdrop, the idea that such brief, self-directed interactions could meaningfully increase loneliness or foster emotional dependency seems less plausible than the alternative hypothesis—that individuals who were already experiencing distress were more likely to seek out and engage with the chatbot more frequently.

### Interpreting the effect sizes

Finally, it is worth considering the magnitude of the reported effects. Across outcomes, the effect sizes in this study were uniformly small—often so small that they are unlikely to translate into meaningful real-world implications. For example, in Table 6 discussed above, the strongest reported interaction effect—the moderating effect of non-personal conversation topics on socialization—was *β* = 0.05, which corresponds to an *r* of approximately 0.05. Other outcomes, such as emotional dependence and problematic use, yielded similar or even smaller coefficients. These findings do not negate the value of the research, but they do urge caution in drawing strong conclusions about practical or clinical significance at this early stage.

### Caution in interpreting absence of harm

While our critique has focused on methodological limitations that complicate conclusions about the potential harms of AI-based therapy chatbots, it is equally important to emphasize that these same limitations also restrict any strong inferences about their safety.

First, the four-week duration of the study, although practical for an early-stage trial, is insufficient to capture potential long-term psychosocial outcomes, such as ingrained dependency or sustained reductions in offline socialization. Second, the relatively short average daily usage time of 5.32 min leaves open the possibility that higher or more prolonged exposure could lead to different effects, whether beneficial or adverse. Finally, although our analysis suggests that reverse causality is a plausible explanation for the observed correlation between higher chatbot use and negative outcomes, we do not dismiss the possibility of differential risks. Vulnerable subgroups, such as individuals experiencing significant distress, may respond differently to AI-based interactions and could be disproportionately susceptible to adverse effects.

These caveats highlight the need for caution in interpreting not only the original study by MIT and OpenAI, but also our own critique of its findings. While we challenge strong claims of harm based on the available data, we also acknowledge that the absence of clear evidence for harm should not be mistaken for evidence of safety.

## Discussion

The study by Fang et al. raises important and timely questions about the psychological effects of interacting with AI-based therapy chatbots. While the authors should be commended for their rigorous and innovative approach, our analysis suggests that the findings—particularly those related to negative psychosocial outcomes—should be interpreted with caution. Several methodological limitations, including challenges in defining appropriate control conditions and assessing usage effects, complicate strong causal claims and limit the conclusions that can be drawn about both harm and safety.

In our view, the current results do not substantiate concerns about increased loneliness or emotional overdependence. As with every new technology, concerns—both valid and overstated—are to be expected. In previous work, we have examined similar dynamics in the context of screen use, where fears about harm were often amplified despite limited empirical support [e.g., (18, 19)]. To avoid repeating this Sisyphean cycle of technological moral panic (20), we must resist the urge to adopt prematurely alarmist narratives around “AI addiction” (21).

At the same time, we should be careful not to mistake the absence of clear evidence for harm as confirmation of safety. It remains important to stay alert to early signs of overreliance on AI therapy bots, particularly among vulnerable populations. We therefore recommend continued investment in careful, incremental studies—such as the one reviewed here—that can guide responsible, evidence-based implementation.

Future research should include (1) experimental manipulations of usage duration to assess its causal influence on psychosocial outcomes, (2) outcome measures based on changes over time, (3) more clearly defined placebo conditions, and (4) comparison groups receiving conventional psychological treatment. Importantly, even well-established therapeutic modalities may sometimes aggravate symptoms or fail to help certain patients (22). Yet these risks are not a reason to abandon traditional therapy altogether—they are part of the broader reality of mental health care. The same perspective should guide our approach to emerging AI-based interventions.

Rather than expecting perfection from new technologies, we should aim for progress—grounded in evidence, attentive to risks, and open to innovation. In the face of a growing mental health crisis, the question, in our view, should not be whether AI is perfect—but whether we are willing to explore its promise responsibly, with both courage and care.

## Data Availability

The original study reviewed in this commentary is publicly available at: https://www.media.mit.edu/publications/how-ai-and-human-behaviors-shape-psychosocial-effects-of-chatbot-use-a-longitudinal-controlled-study/.
