# Correction: Balancing promise and concern in AI therapy: a critical perspective on early evidence from the MIT–OpenAI RCT

**DOI:** 10.3389/fmed.2025.1643202

**Published:** 2025-06-24

**Authors:** Yaakov Ophir, Refael Tikochinski, Zohar Elyoseph, Yaniv Efrati, Hananel Rosenberg

**Affiliations:** ^1^Department of Education, Ariel University, Ariel, Israel; ^2^Centre for Human-Inspired Artificial Intelligence (CHIA), University of Cambridge, Cambridge, United Kingdom; ^3^Experimental Psychology Department, University College London, London, United Kingdom; ^4^Faculty of Education, University of Haifa, Haifa, Israel; ^5^Faculty of Education, Bar-Ilan University, Ramat Gan, Israel; ^6^The Moscowitz School of Communication, Ariel University, Ariel, Israel

**Keywords:** AI therapy, digital mental health, technological panic, mental health innovation, AI in mental health

Affiliation [The Moscowitz School of Communication, Ariel University, Ariel, Israel] was omitted for author [Hananel Rosenberg].

Author [Hananel Rosenberg] was erroneously assigned to affiliation [Department of Education, Ariel University, Ariel, Israel].

Affiliation [Centre for Human-Inspired Artificial Intelligence (CHIA), University of Cambridge, Cambridge, England, United Kingdom] was erroneously given as [Department of Education, University of Cambridge, Cambridge, England, United Kingdom].

Affiliation [Experimental Psychology Department, University College London, London, England, United Kingdom] was erroneously given as [Faculty of Education, University College London, London, England, United Kingdom].

The original article has been updated.

